# Zorrimidazolone, a Bioactive Alkaloid from the Non-Indigenous Mediterranean Stolidobranch *Polyandrocarpa zorritensis*

**DOI:** 10.3390/md9061157

**Published:** 2011-06-23

**Authors:** Anna Aiello, Ernesto Fattorusso, Concetta Imperatore, Carlo Irace, Paolo Luciano, Marialuisa Menna, Rita Santamaria, Rocco Vitalone

**Affiliations:** 1 The NeaNat Group, Department of Chemistry of Natural Products, University of Napoli “Federico II”, Via D. Montesano 49, Napoli 80131, Italy; E-Mails: aiello@unina.it (A.A.); fattoru@unina.it (E.F.); cimperat@unina.it (C.I.); rocco.vitalone@unina.it (R.V.); 2 Department of Experimental Pharmacology, University of Napoli “Federico II”, Via D. Montesano 49, Napoli 80131, Italy; E-Mail: carlo.irace@unina.it (C.I.); rita.santamaria@unina.it (R.S.); 3 C.S.I.A.S. (Interdepartmental Service Center for Spectroscopic Analysis), University of Napoli “Federico II”, Via D. Montesano 49, Napoli 80131, Italy; E-Mail: pluciano@unina.it

**Keywords:** ascidians, natural products, alkaloids, bioactive molecules, cytotoxic activity

## Abstract

Chemical analysis of the Mediterranean ascidian *Polyandrocarpa zorritensis* (Van Name 1931) resulted in the isolation of a series of molecules including two monoindole alkaloids, 3-indolylglyoxylic acid (**3**) and its methyl ester (**4**), 4-hydroxy-3-methoxyphenylglyoxylic acid methyl ester (**1**) and a new alkaloid we named zorrimidazolone (**2**). The structure of the novel compound **2** has been elucidated by spectroscopic analysis and bioactivity of all compounds has been investigated. Zorrimidazolone (**2**) showed a modest cytotoxic activity against C6 rat glioma cell line.

## 1. Introduction

Colonial ascidians are one of the most important marine bio-resources for new bioactive substances and this justifies the considerable interest shown by chemists and pharmacologists in this class of marine invertebrates. Moreover, a comprehensive understanding of their secondary metabolites and/or their distribution among species could give further precious information. It could shine a light, for example, on the role of these metabolites in survival and adaptation of the invertebrates in diverse habitats as well as on the influence of the presence of chemically defended species on the development of benthic marine communities. As a part of our continuing search for new bioactive compounds from Mediterranean ascidians, we have investigated the stolidobranch ascidian *Polyandrocarpa zorritensis* (Van Name 1931). This is a non-indigenous ascidian in the Mediterranean Sea; it was indeed originally discovered and described in Peru and, later found in the northern Mediterranean in the summer of 1974 [1]. Subsequently, in 1986, the species was found in the eastern Mediterranean [2] and, more recently (June 2001), in the harbor of Taranto (South Italy), where colonies developed vigorously on all hard substrata in shallow water and now represent one of the most important elements of the local fouling community [3]. We have now examined specimens of *P. zorritensis* collected in the bay of Taranto. This study resulted in the isolation of four compounds: 4-hydroxy-3-methoxyphenylglyoxylic acid methyl ester (**1**), a new alkaloid named by us as zorrimidazolone (**2**), 3-indolylglyoxylic acid (**3**) and its methyl ester (**4**). The new metabolite **2** belongs to the 2-aminoimidazolone class of marine metabolites, which have been predominantly isolated from *Axinella* and *Agelas* marine sponges and very rarely found in the ascidians. The compounds from this class isolated from ascidians are the *N*,*N*-dimethylaminoimidazolone found in *Dendrodoa grossularia* [4,5] and the polyandrocarpamines A and B in a Fijian *Polyandrocarpa* sp. [6]. 3-Indolylglyoxylic acid (**3**) and 4-hydroxy-3-methoxyphenylglyoxylic acid methyl ester (**1**) have been reported as synthetic intermediates [7,8] but their occurrence in any natural source has never been reported. Compound **4** has also been reported as an intermediate in the synthesis of natural products [9,10] and recently found in the methanol extract of a marine sponge [11]. In fact, compounds **1** and **4** are presumed to be artifacts due to the use of methanol during the isolation procedures.

The effect of compounds **1**–**4** on H9c2 (rat cardiac myoblast), HeLa (cervical cancer), and C6 (rat glioma) cells was investigated by evaluation of cell growth and viability.

## 2. Results and Discussion

### 2.1. Isolation and Structure Elucidation of Compounds **1**–**4**

Freeze-dried specimens of *P. zorritensis* were exhaustively extracted at room temperature with methanol and, subsequently, with chloroform. Combined extracts were then partitioned between water and ethyl acetate and, subsequently, between water and butanol. Both ethyl acetate and butanol soluble material were subjected to flash chromatography on a normal phase and on a reverse C_18_ bonded silica column, respectively. Subsequent purifications of the obtained fractions by reverse and normal phase HPLCs yielded compounds **1**–**4** in the pure state. Compounds **3** and **4** were easily identified by comparison of their spectroscopic properties with those reported in literature [7,11]. Ester **1**, as well as the corresponding 4-hydroxy-3-methoxyphenylglyoxylic (vanilloylformic) acid, have been very recently reported as intermediates in the preparation of resiniferatoxin analogues [8]. Moreover, early syntheses of vanilloylformic acid are reported [12–14], however no spectroscopic data useful for the identification of **1** were available. The structure of this compound, and new compound **2**, was established by NMR spectroscopic analysis (Table 1).

The ESI mass spectrum (positive ion mode) of compound **1** displayed ion peaks at *m/z* 211, 233, and 249 corresponding to [M + H]^+^, [M + Na]^+^, and [M + K]^+^, respectively. The molecular formula C_10_H_10_O_5_ was established by HRESIMS analysis on the [M + H]^+^ peak (*m/z* = 211.0615, calculated value: 211.0606) and implied six unsaturation degrees. The ^13^C NMR spectrum displayed signals for all the ten carbons, which were identified, on the basis of HSQC data and of their chemical shift values, as two methyls linked to heteroatoms, three sp^2^ methines, three sp^2^ unprotonated carbons, and two carbonyls (see Table 1); these two latter signals accounted for two of the six unsaturation degrees indicated. The ^1^H NMR spectrum (CDCl_3_) of **1** contained three aromatic signals at δ 6.99 (1H, d, *J* = 8.1), 7.59 (1H, d, *J* = 1.7) and 7.61 (1H, dd, *J* = 8.1, 1.7); the presence in **1** of a 1,2,4-trisubstituted benzene ring was thus deduced, on the basis of the coupling constant pattern of the above proton signals and according to ^13^C spectrum data, and the remaining four unsaturations were accounted for. Additional features of the proton spectrum of **1** were two methoxyl signals at δ 3.98 and δ 3.96, and a signal at δ 6.24 (1H, s) exchangeable in D_2_O. The latter signal, attributable to the proton of a phenol function, was correlated in the HMBC spectrum to both the down field shifted unprotonated aromatic carbons at δ 147.0 and 152.2, as well as to the methine carbon at δ 114.3; besides, the methoxyl signal at δ 3.98 showed a long-range coupling with the carbon signal at δ 147.0. Thus, the presence of two oxygenated functions (hydroxyl and methoxyl) *ortho*-orientated on the benzene ring was deduced. The nature of the remaining substituent as well as its location on the aromatic ring was deduced by analysis of HMBC and ROESY data. The methoxyl signal at δ 3.96 was long range coupled to both the carbonyls at δ 164.2 and 184.4; the latter carbonyl signal showed HMBC correlations with the aromatic proton signals at δ 7.59 (H-2′), showing only a small *meta*-coupling, and 7.61 (H-6′), *ortho*-coupled to the signal at δ 6.99 (H-5′). This information suggested an oxoacetic acid methyl ester moiety to be linked at the benzene ring, according to the molecular formula and the nature of the remaining NMR signals. Moreover, a strong correlation observed in the ROESY spectrum between the methoxyl signal at δ 3.98 (MeO-) and the aromatic proton at δ 7.59 (H-2′) was observed and, therefore, the structure of compound **1** was established as methyl 2-(4-hydroxy-3-methoxyphenyl)-2-oxoacetate.

**Figure 1:**
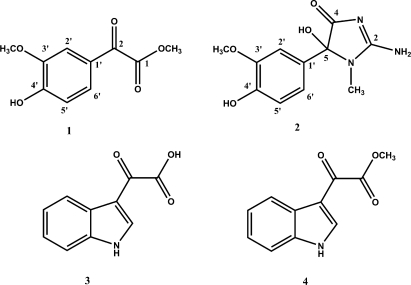
Structures of compounds **1**–**4**.

ESI mass spectrum (positive mode) of zorrimidazolone (**2**) showed ion peaks at *m/z* 252 and 274, corresponding to [M + H]^+^ and [M + Na]^+^, respectively; the molecular formula was shown to be C_11_H_13_N_3_O_4_ by HRESIMS analysis on the [M + H]^+^ peak (*m/z* = 252.0995, calculated value: 252.0984) and indicated seven unsaturation degrees. According to mass data, ^13^C NMR spectrum (CD_3_OD) of **2** contained 11 signals which, on the basis of HSQC data, were sorted as two methyls, three sp^2^ methines, one sp^3^ as well as five sp^2^ quaternary carbons. Proton and ^13^C NMR spectra (CD_3_OD, Table 1), interpreted on the basis of HSQC and HMBC data, revealed the presence in **2** the same 4-hydroxy-3-methoxyphenyl moiety as in **1**, indicating the different structural moiety at C-1′. This portion of the molecule accounted for four unsaturation degrees, leaving three unsaturations for the remaining C_4_H_6_N_3_O_2_ subunit. This unidentified sub-structure contained three quaternary carbons, two of them attributable to carbonyls (δ 170.5 and 189.9), and one N-linked methyl group (δ_C_ 26.3; δ_H_ 2.81); hence, one ring was present in this subunit and the three remaining hydrogen atoms were attached to heteroatoms. In the ^1^H NMR spectrum of **2** recorded in DMSO-*d*_6_ (see Experimental), in addition to the phenol signal at δ 7.01, two exchangeable signal at δ 5.41 and 7.48 were visible. The latter signal was correlated in the ROESY spectrum (DMSO-*d*_6_) to the *N*-methyl signal at δ 2.65. The location of the methyl group at N-1 was deduced by the observation of the correlations between the signal at δ 2.65 and the two aromatic protons at δ 6.84 (H-2′) and 6.55 (H-6′); a weaker correlation to the proton at δ 6.73 (H-5′) was also observed. The chemical shift value (δ 91.9) of the third sp^3^ unprotonated carbon present in this subunit was characteristic of a carbinolamine carbon. On the basis of its HMBC (CD_3_OD) correlations with the aromatic methine protons at δ 7.05 and 6.71, this carbon was assumed to be attached to the aromatic ring. Two further informative correlations were present in the HMBC spectrum (CD_3_OD) of **2** between the N-linked methyl group (δ 2.81) and the carbon resonances at δ 91.9 (C-5) and 170.5 (C-2). The above NMR data as a whole suggested the presence of a 2-amino-5-hydroxy-1-methylimidazolone moiety linked to the 2-methoxyphenol unit, according to mass information and endorsed by comparison with data reported in literature for other aminoimidazolone compounds [5,6]. Thus, the structure of zorrimidazolone (**2**) was defined as reported in Figure 1. However, it should be pointed out that the reported tautomeric form was deduced by NMR experiments recorded in DMSO-*d*6 and that the predominance of another tautomer in different solvents cannot be excluded. In spite of the presence of the stereogenic center at C-5, zorrimidazolone did not show optical rotation either Cotton effects on the CD spectrum (MeOH).

**Figure 2:**
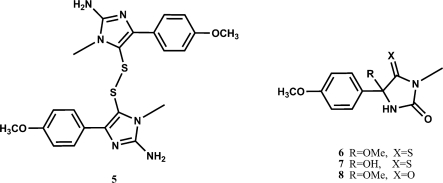
Structures of polycarpine (**5**) and related compounds (**6**–**8**).

Aminoimidazolone ring-containing metabolites, like zorrimidazolone, have been reported from other ascidians, also belonging to *Polyandrocarpa* genus [6]. In particular, compounds structurally related to zorrimidazolone (**6**–**8**) have been isolated from *Polycarpa clavata* and *P. aurata* and they were hypothesized to be artifacts derived from polycarpine (**5**) (Figure 2). In the light of the above report, we considered that zorrimidazolone could derive from a hypothetic parent dimeric disulfide alkaloid, as compounds **6**–**8** from polycarpine (**5**). However, this possibility could be confidently excluded since (i) in the isolation procedure of **2** we didn’t use silica and basic condition which very probably were the causative agent of the degradation of polycarpine; (ii) in the extract of *P. zorritensis* no trace was detected of the putative parent disulfide nor of a thioxoimidazolone compound.

**Scheme 1.**
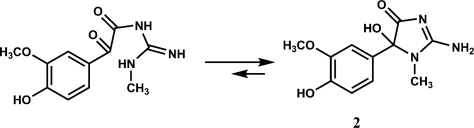
Equilibrium of compound **2** with the guanidinic open form.

Finally, the optical inactivity of **2**, suggesting its racemic nature, is strongly indicative that **2** could be in equilibrium with the guanidinic open form (see Scheme 1). Interestingly, the occurrence of guanidinic derivatives of methoxyphenols from ascidians of the genus *Polycarpa* have been recently reported [15].

### 2.2. Biological Activities of Compounds **1**–**4**

Bioactivity of compounds **1**–**4** was investigated on H9c2 rat cardiac myoblast cells, HeLa cervical cancer cells and C6 rat glioma cells by evaluation of cell growth and viability. To this aim, the cells were treated for 48 h with various concentrations of each compound and then the cell count and the MTT assay were performed. The results, reported in Table 2 as IC_50_ values, showed that, with the exception of compound **1**, compounds **2**, **3** and **4** exhibited a selective and concentration-dependent cytotoxic activity toward the C6 cells, whereas no relevant cytotoxicity was observed against HeLa and H9c2 cells. Zorrimidazolone (**2**) showed the higher cytotoxic effect *versus* C6 cells leading to a decrease of viability of about 60% when administrated at 250 μM; its IC_50_ value against C6 cells was within the micromolar range (∼150 μM), which is generally considered as a moderate cytotoxic activity.

The rat C6 cells line represent a useful *in vitro* model for studying gliomas. Although many advances in antineoplastic therapy have taken place, many malignant gliomas are resistant to many pharmacological treatments [16]. Therefore, zorrimidazolone could be of interest in the developing of potential gliomas antiproliferative molecules.

## 3. Experimental Section

### 3.1. General Experimental Procedures

ESI mass spectra were recorded on a hybrid quadrupole-TOF mass spectrometer in MeOH. The spectra were recorded by infusion into the ESI source using MeOH as the solvent. HRESIMS (positive mode) were performed on a Thermo LTQ Orbitrap XL mass spectrometer. Optical rotations were measured with a Perkin-Elmer 192 polarimeter at 589 nm using a 10 cm microcell. ECD spectra were recorded on a J-710 spectropolarimeter (Jasco, Tokyo, Japan) equipped with a J-710 for Windows software (Jasco). ^1^H (700 MHz and 500 MHz) and ^13^C (175 MHz and 125 MHz) NMR spectra were recorded on a Varian INOVA spectrometer; chemical shifts were referenced to the residual solvent signal (methanol-*d*_4_: δ_H_ 3.31, δ_C_ 49.0; chloroform-*d*: δ_H_ 7.26, δ_C_ 77.0). Homonuclear ^1^H connectivities were determined by COSY and TOCSY (mixing time 100 ms) experiments. Through-space ^1^H connectivities were evidenced using a ROESY experiment with a mixing time of 500 ms. Two and three bond ^1^H-^13^C connectivities were determined by gradient 2D HMBC experiments optimized for a *J*_2,3_ of 8 Hz.

### 3.2. Collection, Extraction and Isolation

Specimens of *P. zorritensis* were collected in the bay of Taranto and kept frozen until used. Freshly thawed organisms (47.078 g dry weight after extraction) were exhaustively extracted at room temperature with methanol (3 × 1.5 L) and, subsequently, with chloroform (3 × 1.5 L). The extracts were combined and concentrated *in vacuo*; the resulting aqueous residue was then partitioned between water and ethyl acetate. The organic layer was removed and the water soluble portion was further partitioned in butanol. The ethyl acetate soluble material (3.42 g after solvent evaporation) was subjected to a gradient silica gel MPLC (hexane→AcOEt→MeOH). The 50% Hexane/50% AcOEt fraction was evaporated to dryness under reduced pressure and chromatographed by HPLC on a silica column (LUNA 5 μm, 250 × 4.60 mm), using 70% Hexane/30% AcOEt as the eluent at a flow rate of 0.8 mL/min. This afforded pure compound 1 (2.1 mg) and compound 4 (1.7 mg).

Separation of the butanol soluble material (7.77 g after solvent evaporation) was achieved by a C18 bonded silica MPLC eluting with a step gradient of MeOH in H_2_O from 10% to 100%. The fast-running fraction, eluted with 90% H_2_O/10% MeOH, was first subjected to a preparative reverse phase HPLC on a Kromasil 10 μm column (250 × 10 mm), eluting with 95% H_2_O/5% MeOH at a flow rate of 2 mL/min, to give a mixture of zorrimidazolone and small amounts of other polar metabolites. The mixture was further purified by HPLC on a Synergie 4 μ POLAR-RP 80A column (250 × 4.60 mm) using 98% H_2_O/2% MeOH as the eluent at a flow rate of 1 mL/min; this afforded zorrimidazolone in pure form (1.1 mg).

The MeOH 100% fraction was separated by reverse phase HPLC (LUNA 3 μm, C18, 150 × 4.60 mm), using 65% H_2_O/35% MeOH as the eluent at a flow rate of flow 0.4 mL/min); this yielded compound **3** in pure form (1.5 mg).

### 3.3. Methyl 2-(4-Hydroxy-3-methoxyphenyl)-2-oxoacetate (**1**)

Yellow amorphous powder. HRESIMS (positive ion mode): *m/z* = 211.0615 [M + H]^+^, the molecular formula C_10_H_11_O_5_ requires 211.0606. ^1^H and ^13^C NMR data (CDCl_3_): see Table 1. ^1^H NMR data (DMSO-*d*_6_) 7.43 (overlapped, 1H, H-2′), 6.95 (d, 1H, *J* = 8.1 Hz, H-5′), 7.45 (overlapped, 1H, H-6′), 3.84 (s, 3H, -OCH_3_), 3.91 (s, 3H, -CO_2_CH_3_), 10.6 (s, 1H, -OH).

### 3.4. Zorrimidazolone (**2**)

Pink amorphous powder. HRESIMS (positive ion mode): *m/z* = 252.0995 [M + H]^+^, the molecular formula C_11_H_14_N_3_O_4_ requires 252.0984; ^1^H and ^13^C NMR data (CD_3_OD): see Table 1. ^1^H NMR data (DMSO-*d*_6_): 6.84 (d, 1H, *J* = 1.8 Hz, H-2′), 6.73 (d, 1H, *J* = 8.2 Hz, H-5′), 6.55 (dd, 1H, *J* = 1.8–8.2 Hz, H-6′), 3.74 (s, 3H, -OCH_3_), 2.65 (s, 3H, -NCH_3_), 7.01 (s, 1H, -OH-4′), 5.41 (s, 1H, -OH-5), 7.48 (s, 2H, -NH_2_).

### 3.5. Biological Activity

Biological activity of the isolated compounds was investigated on H9c2 rat cardiac myoblast cells, HeLa cervical cancer cells and C6 rat glioma cells by evaluation of cell growth and viability. Cells were grown in Dulbecco’s modified Eagle’s medium (DMEM) containing high glucose (4.5 g/L) and supplemented with 10% fetal bovine serum (FBS), l-glutamine (2 mM), penicillin (100 units/mL) and streptomycin (100 μg/mL) according to ATCC recommendations. Cells were cultured at 37 °C in a humidified 5% CO_2_ atmosphere. For experimental purposes, cells were washed, collected by trypsine and then inoculated in a 96-microwell culture plates at density of 10^4^ cells/well. Cells were allowed to grow for 24 h, then the medium was replaced with fresh medium and cells were treated for further 48 h with test compounds. In detail, 1 or 2 μL of DMSO solutions containing the test compounds were added to cell culture medium to give various concentration; 1 or 2 μL of DMSO alone (vehicle) were added into control cells (0.5 and 1% v/v final concentrations, respectively).

Cell viability was determined by the trypan blue dye exclusion test. After treatments, the medium was removed and the cells were washed twice with PBS buffer solution and then incubated with a trypsin-EDTA solution at 37 °C for 5 min. Trypsin was inactivated by re-suspending the cells in medium containing 10% FBS. The cells were pelleted at 250× *g* and resuspended in PBS. Viable cells, cells that excluded 0.4% trypan blue, were then counted with a hemocytometer.

Concurrently, cell viability was evaluated with an MTT assay procedure, which measures the level of mitochondrial dehydrogenase activity using 3-(4,5-dimethyl-2-thiazolyl)-2,5-diphenyl-2*H*-tetrazolium bromide (MTT) as substrate [17]. The assay was based on the redox ability of living mitochondria to convert dissolved MTT into insoluble formazan. Briefly, after treatments with the test compounds, the medium was removed and the cells were incubated with 20 μL/well of an MTT solution (5 mg/mL) for 1 h in a humidified 5% CO_2_ incubator at 37 °C. The incubation was stopped by removing the MTT solution and adding 100 μL/well of DMSO to solubilize the formazan. The absorbance was monitored at 550 nm by using a Perkin-Elmer LS 55 Luminescence Spectrometer (Perkin-Elmer Ltd., Beaconsfield, UK) [18].

The calculation of the concentration required inhibiting the net increase in the 48 h cell count and viability by 50% (IC_50_) is based on plots of data carried out in triplicates and repeated three times. IC_50_ values were obtained using a dose response curve by nonlinear regression using a curve fitting program, GraphPad Prism 5.0, and are expressed as mean ± SEM.

## Figures and Tables

**Table 1:** ^1^H and ^13^C NMR data of compounds **1** and **2**.

**Pos.**	**1 ^a^**		**2 ^b^**	

	**δ_H_(mult.,*J*in Hz)**	**δ_C_**	**δ_H_(mult.,*J*in Hz)**	**δ_C_**
**1**		164.2		
**2**		184.4		170.5
**4**				189.9
**5**				91.9
**1′**		125.3		129.2
**2′**	7.59 (d, *1.7*)	110.6	7.05 (d, *1.7*)	110.9
**3′**		147.0		149.1
**4′**		152.2		148.2
**5′**	6.99 (d, *8.1*)	114.3	6.79 (d, *8.1*)	116.1
**6′**	7.61 (dd *8.1*, *1.7*)	126.8	6.71 (dd *8.1*, *1.7*)	119.3
**10**		-		
**11**		-		
**-OC*H*_3_**	3.98 (s)	56.1	3.67 (s)	56.3
**-CO_2_C*H*_3_**	3.96 (s)	52.6		-
**-N*M*e**		-	2.81 (s)	26.3
**-OH**	6.24 (s)			

a Data recorded in CDCl_3_;

b Data recorded in CD_3_OD.

**Table 2:** C6 (rat glioma) cells growth inhibition by compounds **1**–**4**.

**Compound**	**IC_50_ (μM) ^a^**
**1**	>10^3^
**2**	155 ± 13
**3**	314 ± 17
**4**	305 ± 15

a IC_50_ values are expressed as mean ± SEM of three independent experiments conducted in triplicates.
