# Supplementary material for: Zorrimidazolone, a Bioactive Alkaloid from the Non-Indigenous Mediterranean Stolidobranch Polyandrocarpa zorritensis
Source: Mar Drugs. 2011 Jun 23;9(6):1157–65. doi: 10.3390/md9061157 (PMC3131566; doi:10.3390/md9061157)

Supporting Information

^{1}H-NMR spectrum of compound **1** in $CDCl_3$	S2
COSY spectrum of compound **1** in $CDCl_3$	S3
HSQC spectrum of compound **1** in $CDCl_3$	S4
HMBC spectrum of compound **1** in $CDCl_3$	S5
^{13}C-NMR spectrum of compound **1** in $CDCl_3$	S6
^{1}H-NMR spectrum of compound **1** in DMSO	S7
COSY spectrum of compound **1** in DMSO	S8
HSQC spectrum of compound **1** in DMSO	S9
HMBC spectrum of compound **1** in DMSO	S10
ROESY spectrum compound **1** in DMSO	S11
^{1}H-NMR spectrum of zorrimidazolone (**2**) in CD3OD	S12
^{13}C-NMR spectrum of zorrimidazolone (**2**) in CD3OD	S13
HSQC spectrum of zorrimidazolone (**2**) in CD3OD	S14
HMBC spectrum of zorrimidazolone (**2**) in CD3OD	S15
^{1}H-NMR spectrum of of zorrimidazolone (**2**) in DMSO	S16
ROESY spectrum zorrimidazolone (**2**) in DMSO	S17
ESI mass spectrum of compound **1** (**3**)	S18
ESI mass spectrum of zorrimidazolone (**2**)	S19

^{1}H-NMR spectrum of compound **1** in $CDCl_3$

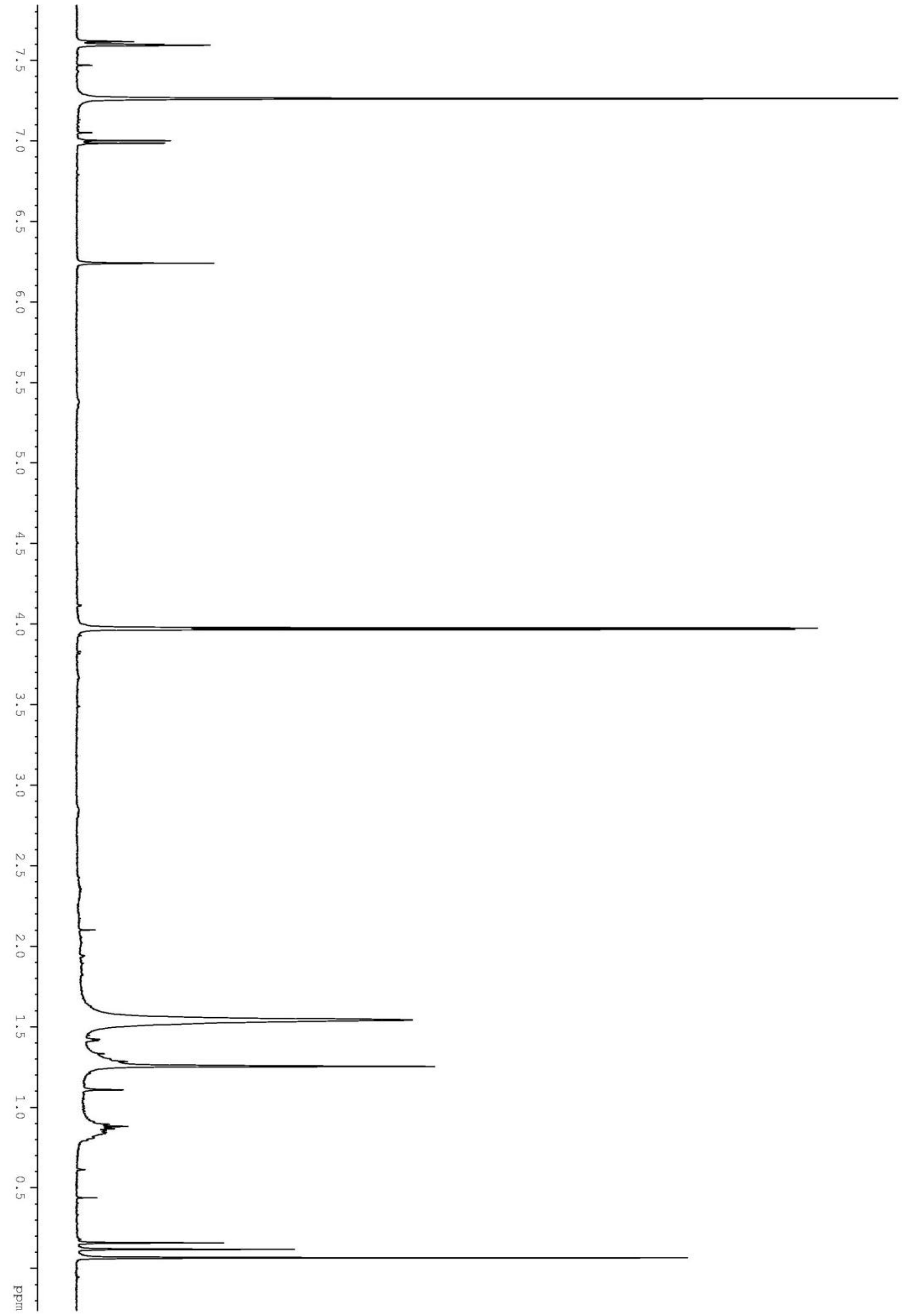

COSY spectrum of compound **1** in $CDCl_3$

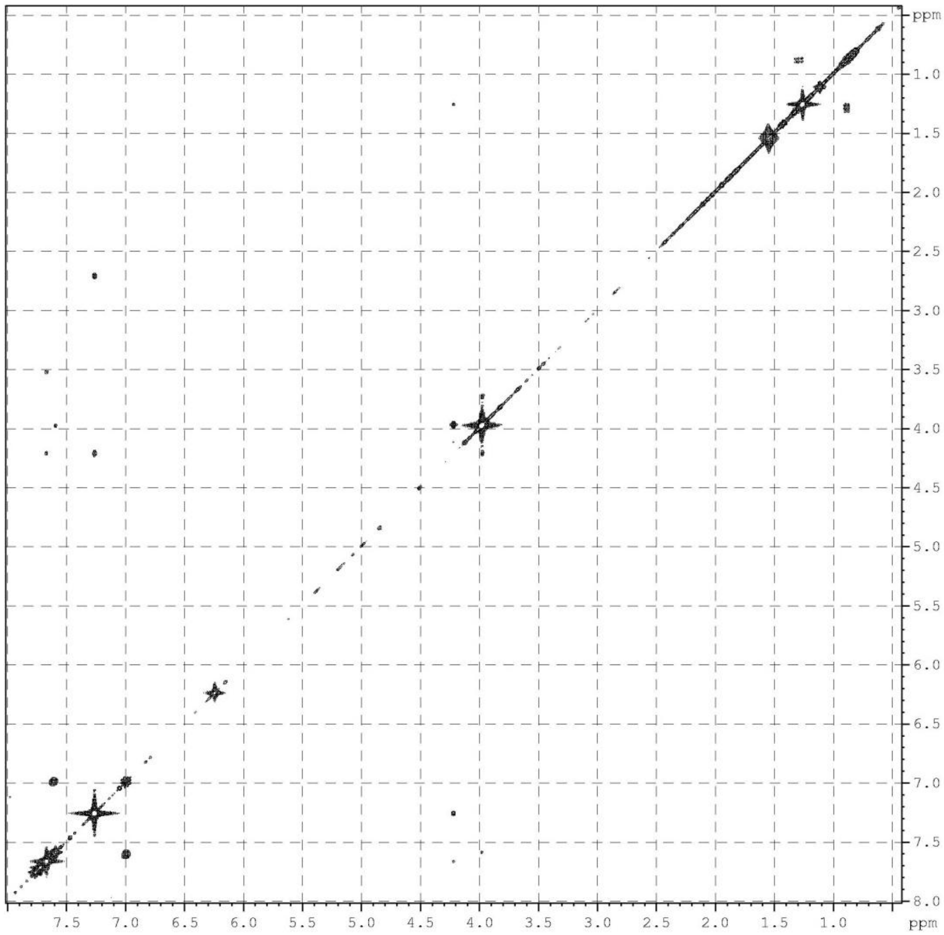

HSQC spectrum of compound **1** in $CDCl_3$

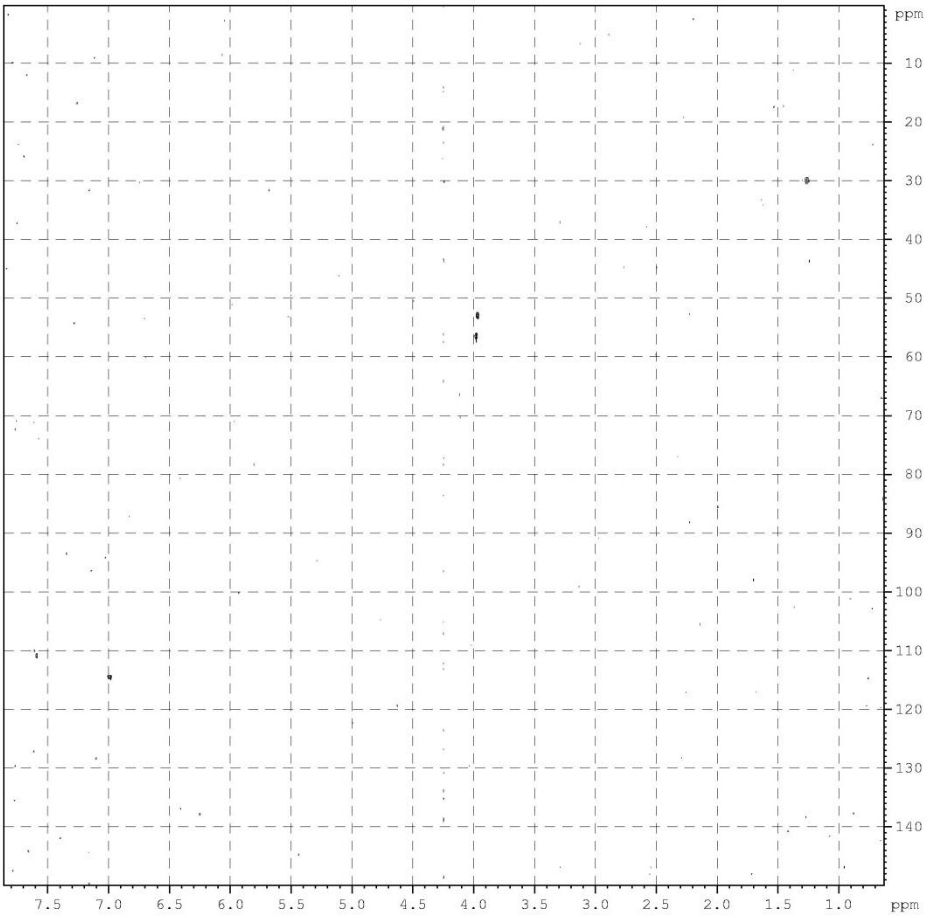

HMBC spectrum of compound **1** in $CDCl_3$

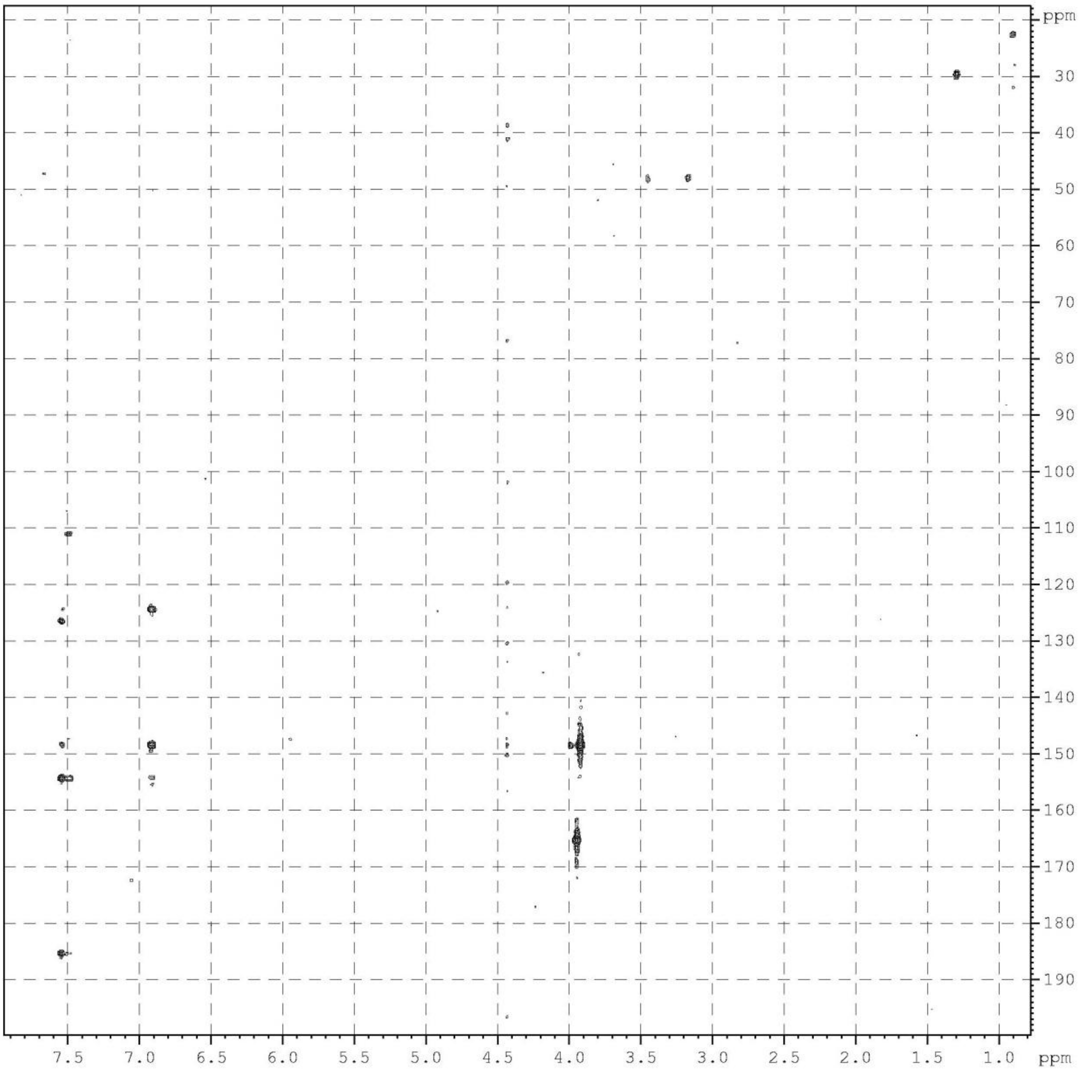

^{13}C-NMR spectrum of compound **1** in $CDCl_3$

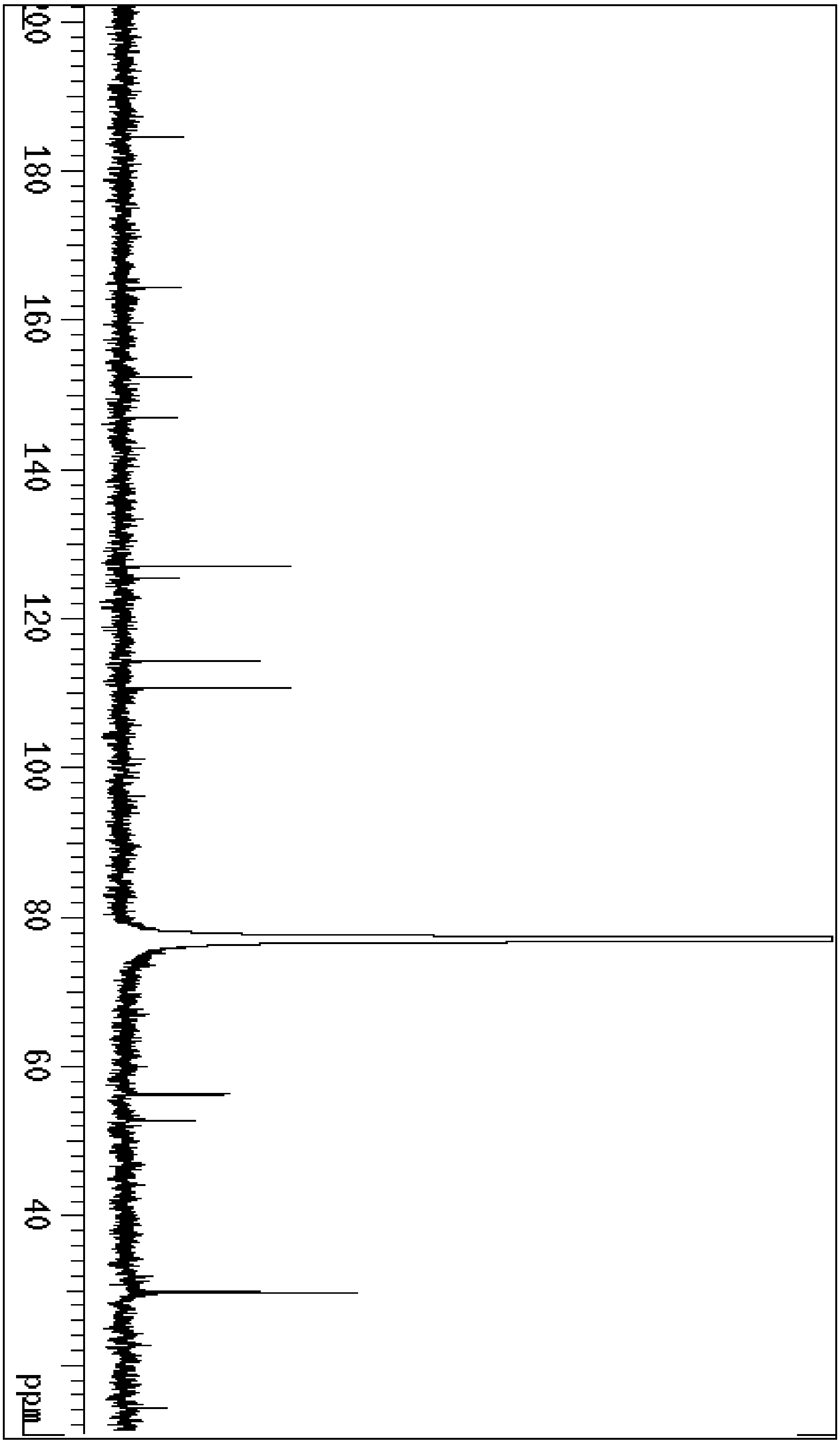

^{1}H-NMR spectrum of compound **1** in DMSO

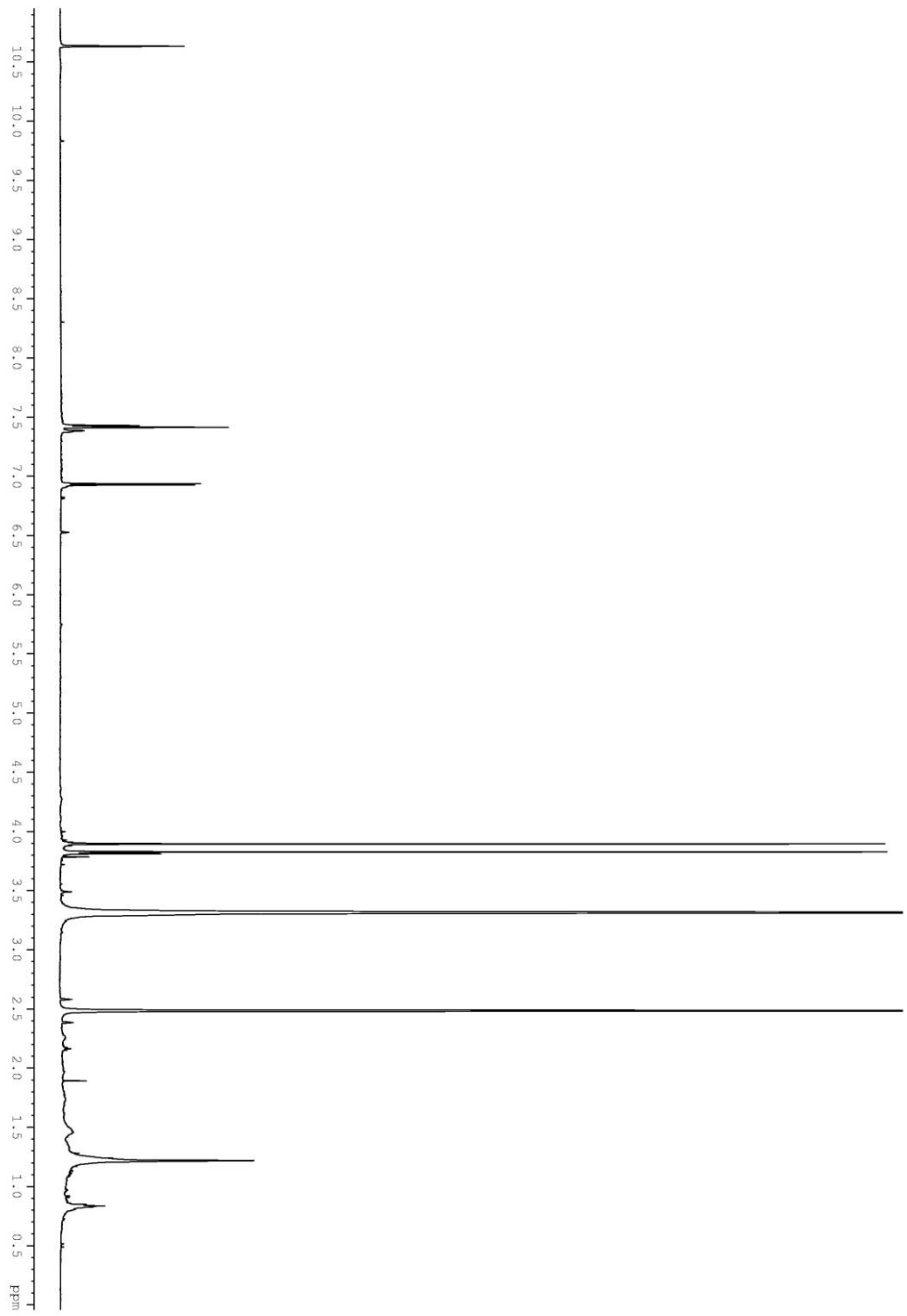

COSY of compound **1** in DMSO

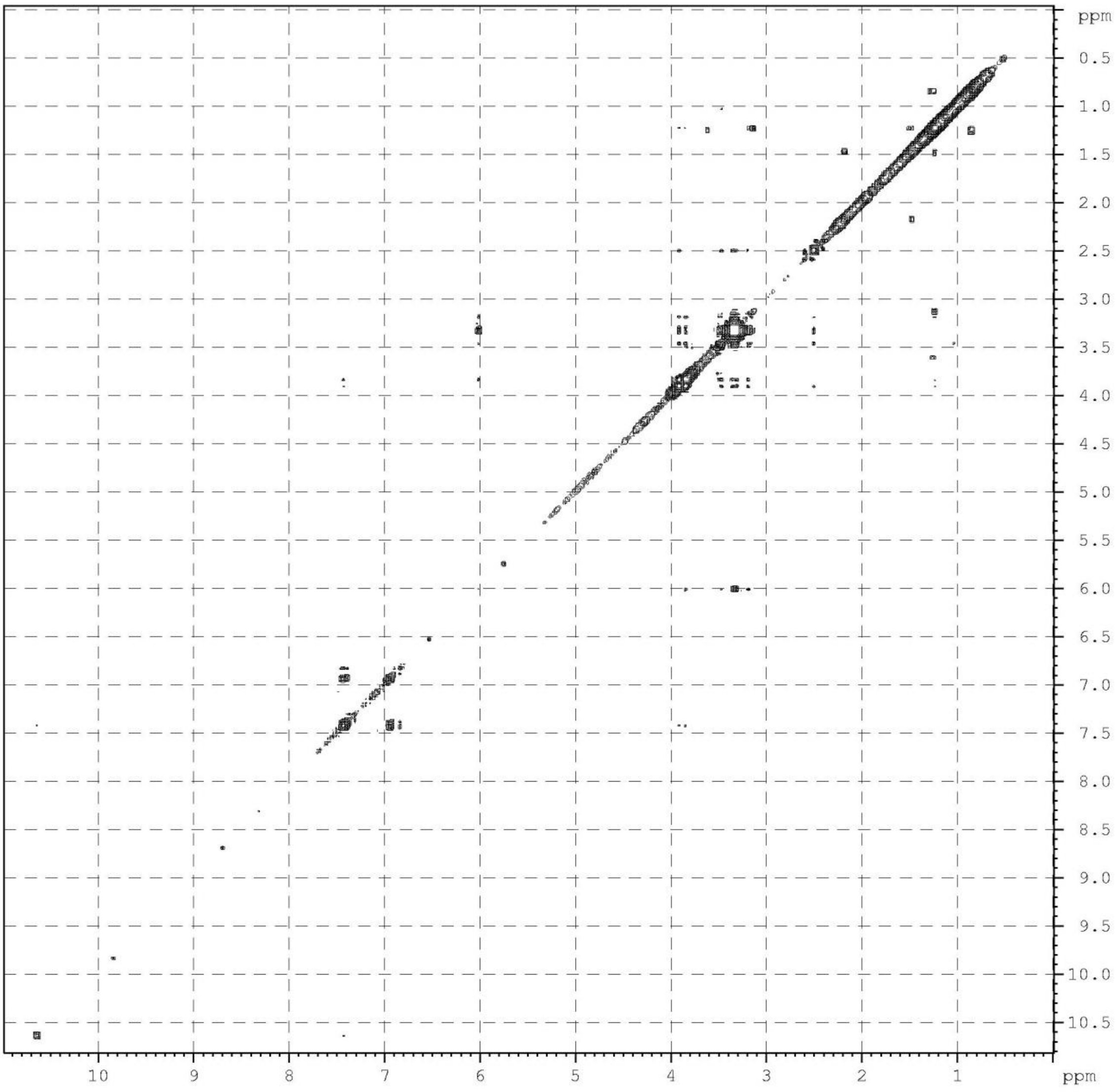

HSQC of compound **1** in DMSO

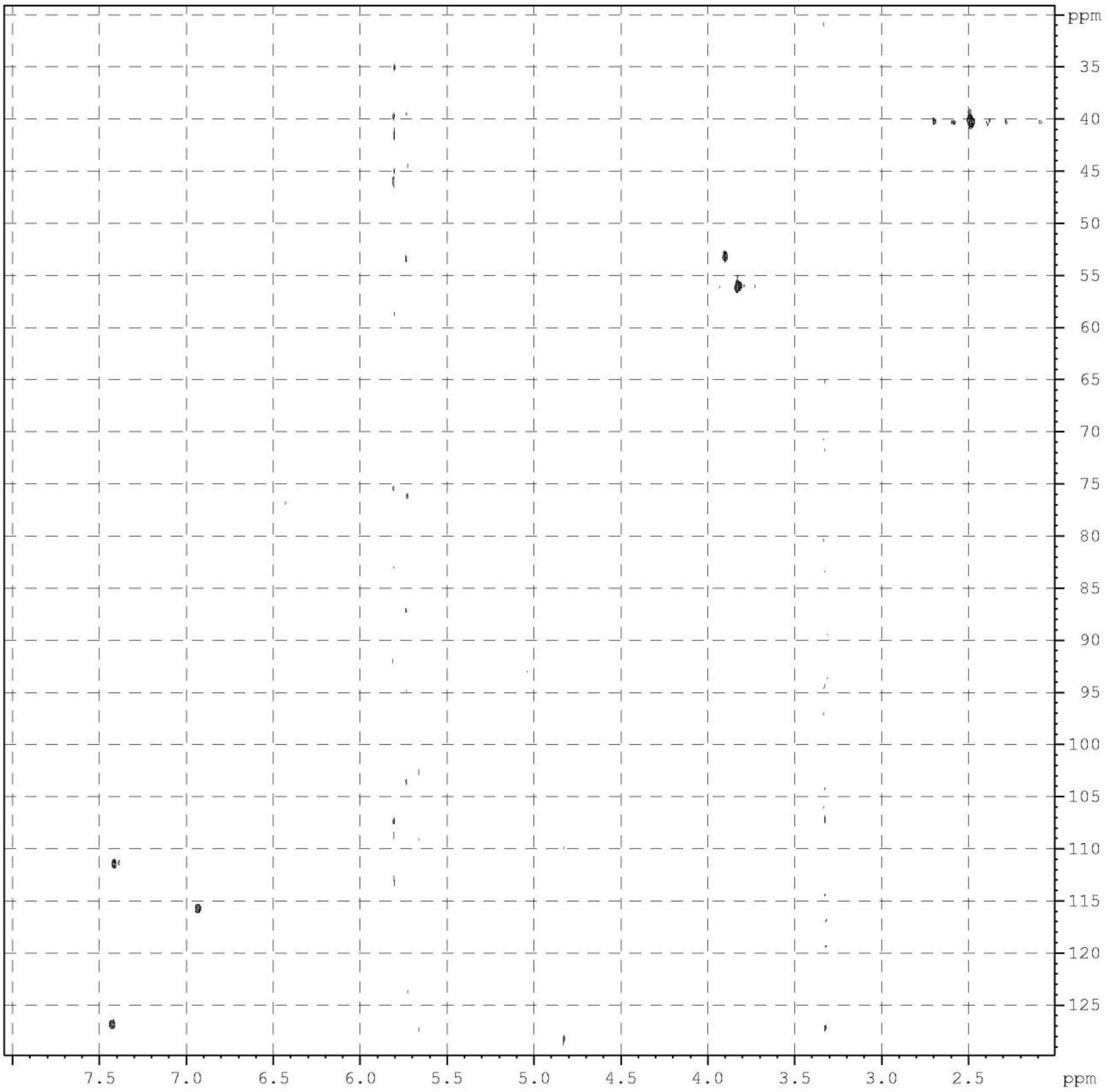

HMBC of compound **1** in DMSO

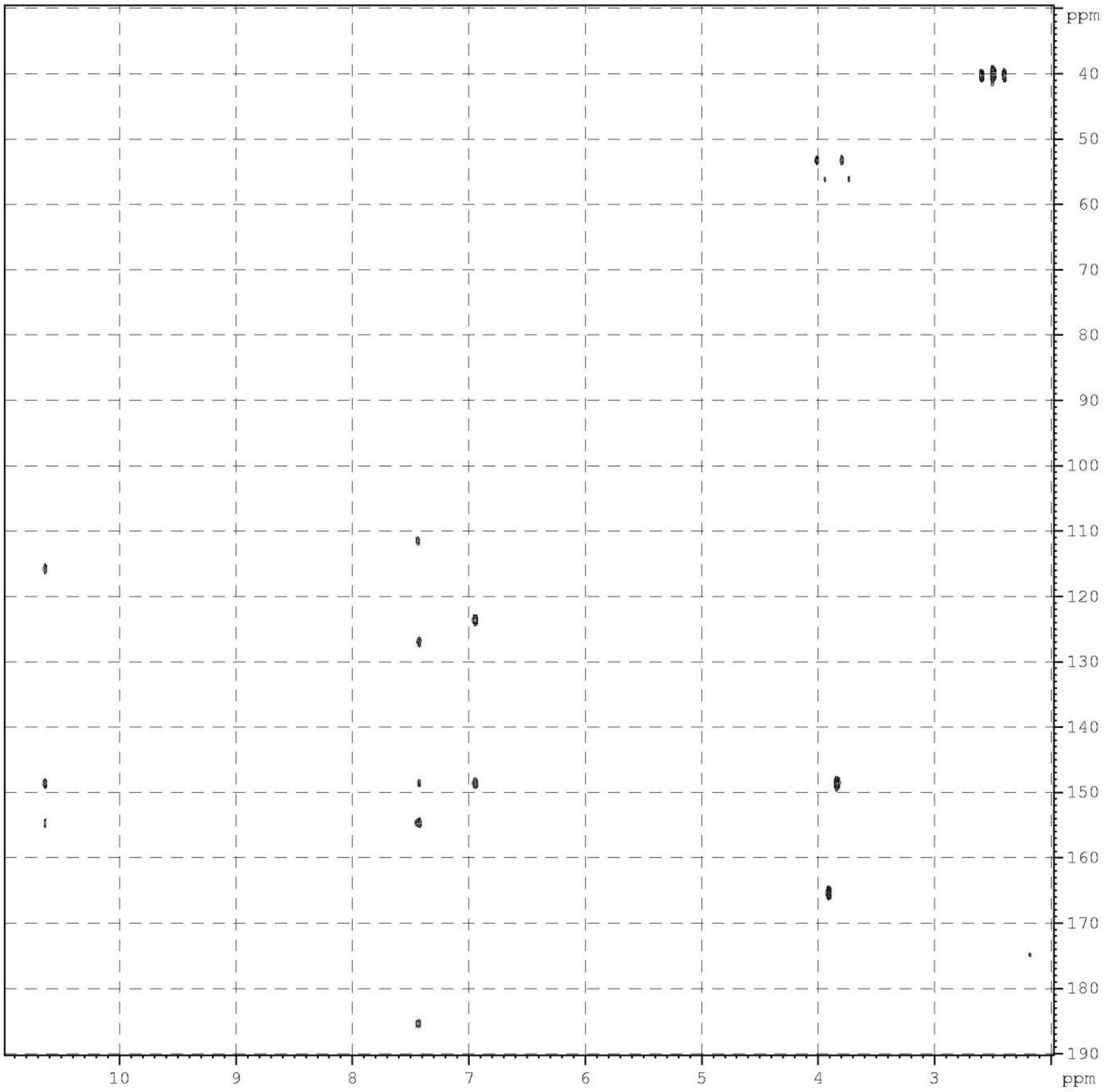

ROESY of compound **1** in DMSO

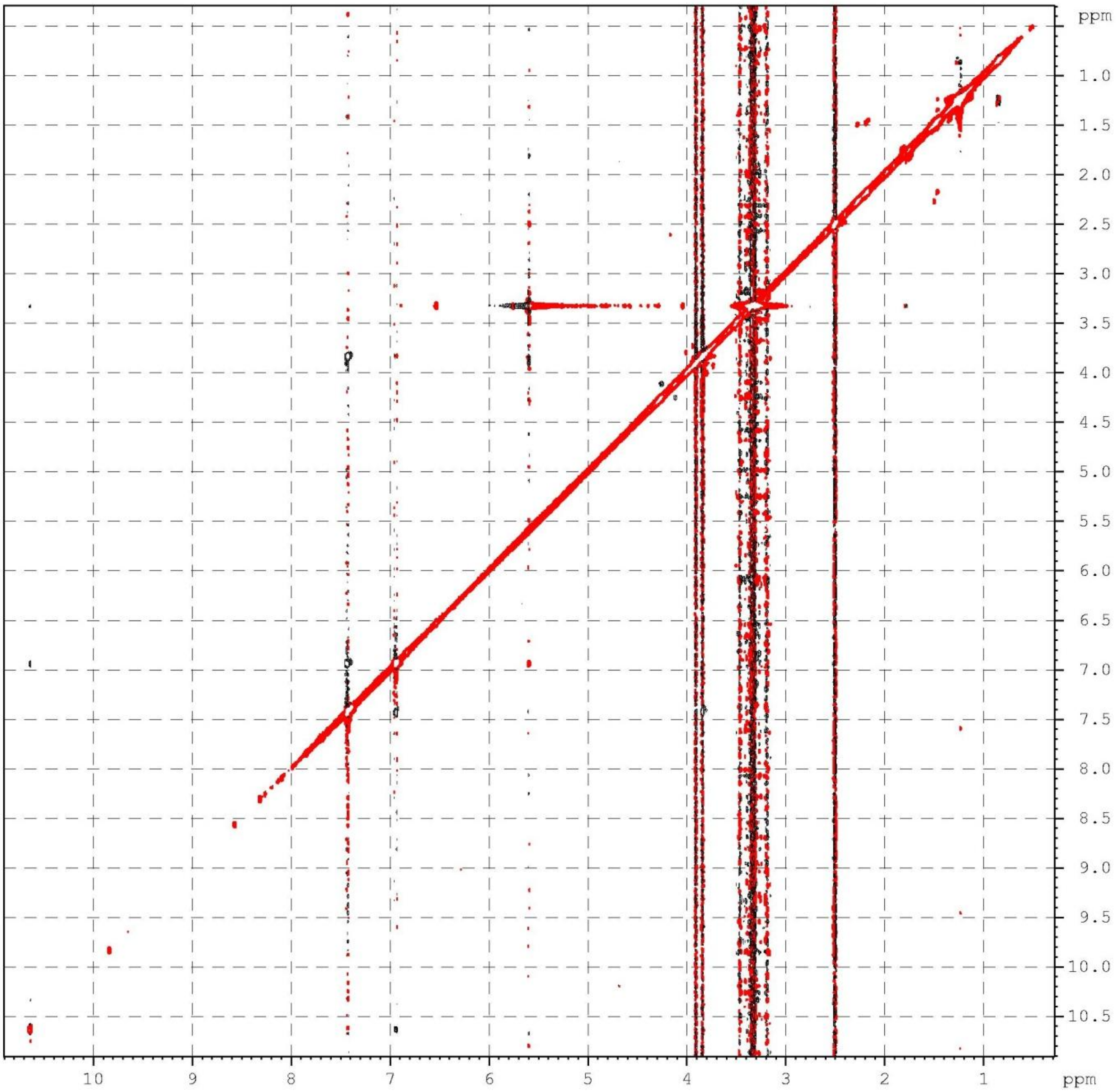

^{1}H-NMR spectrum of zorrimidazolone (**2**) in CD_3OD

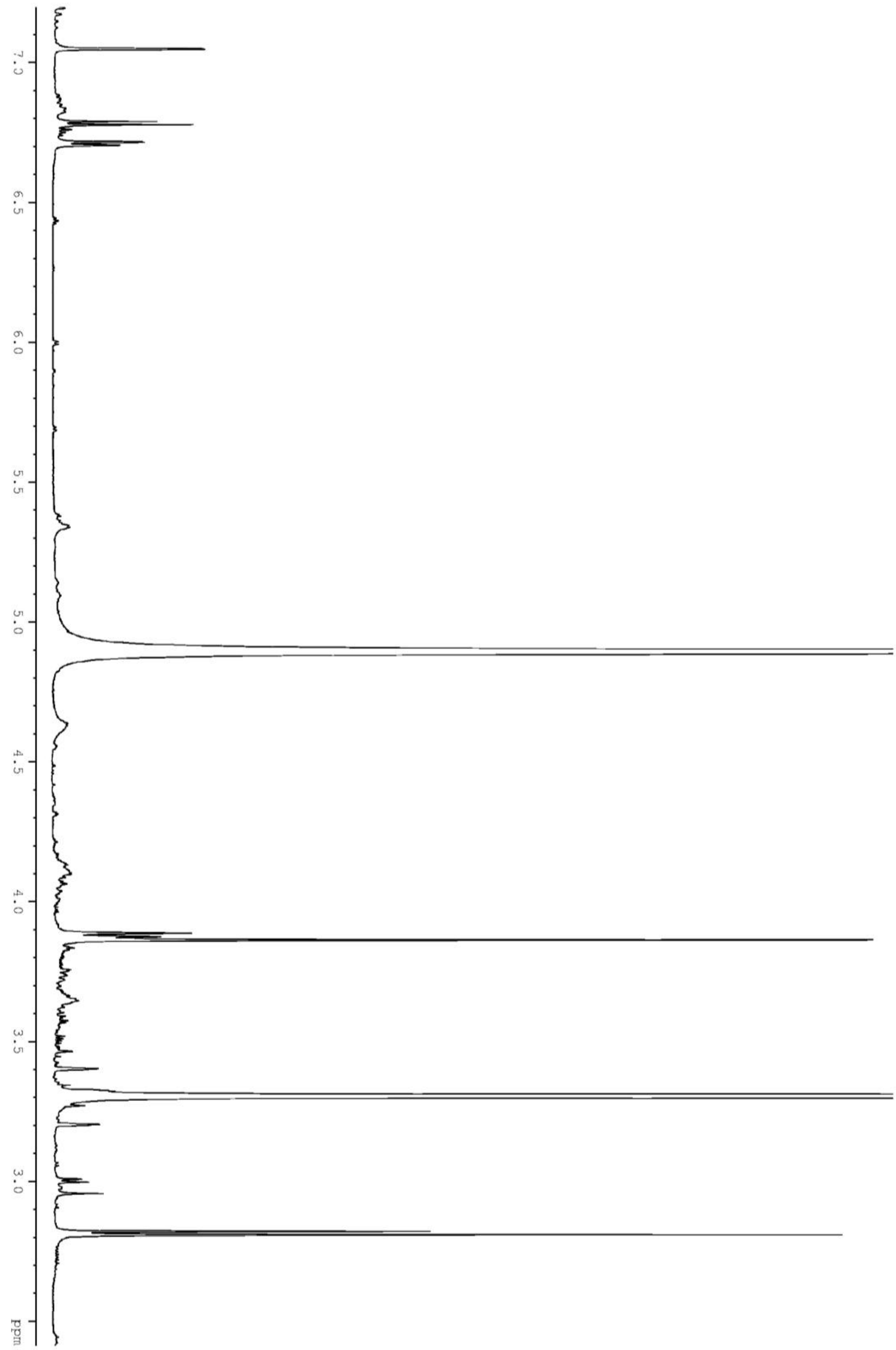

^{13}C-NMR spectrum of zorrimidazolone (**2**) in CD_3OD

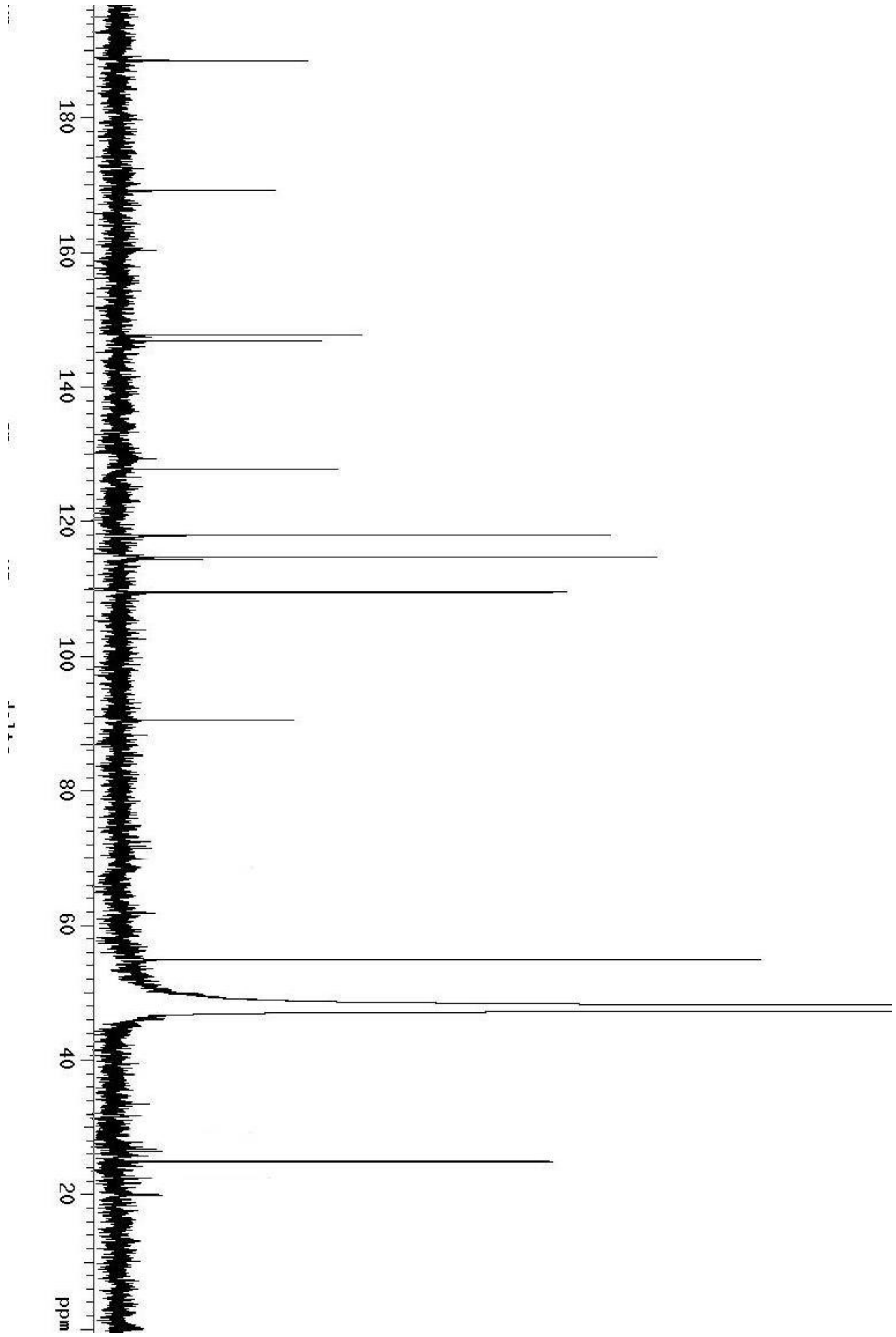

HSQC spectrum of zorrimidazolone (**2**) in CD_3OD

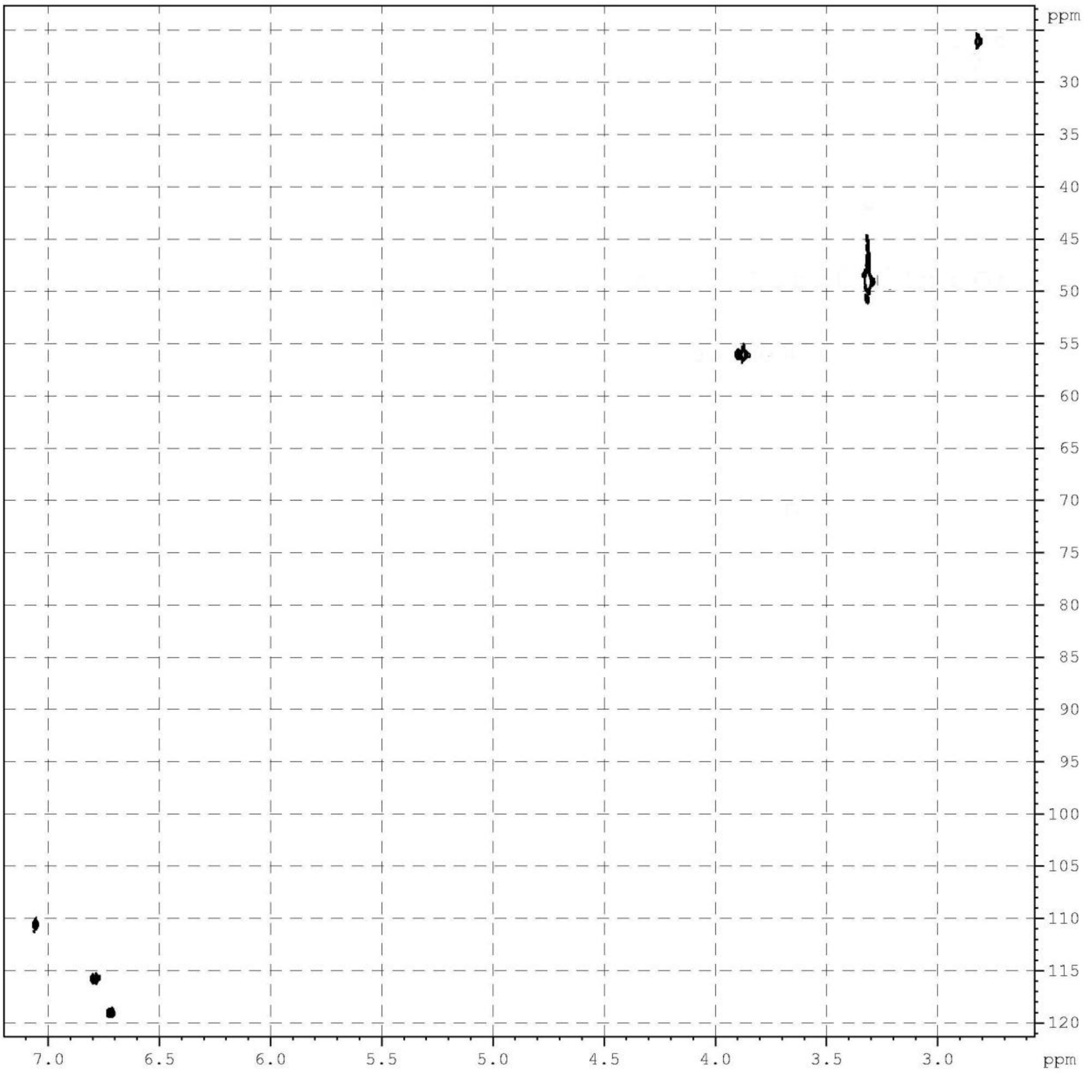

HMBC spectrum of zorrimidazolone (**2**) in CD_3OD

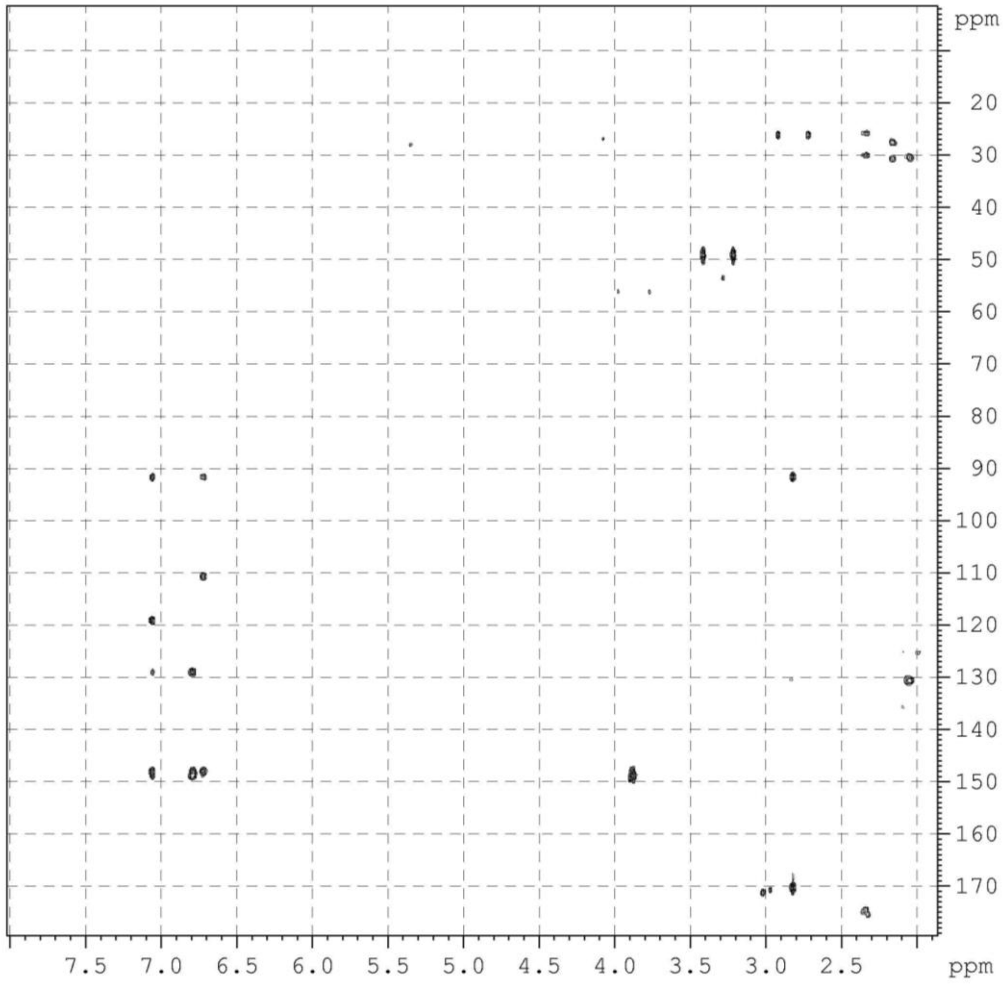

^{1}H-NMR spectrum of zorrimidazolone (**2**) in DMSO

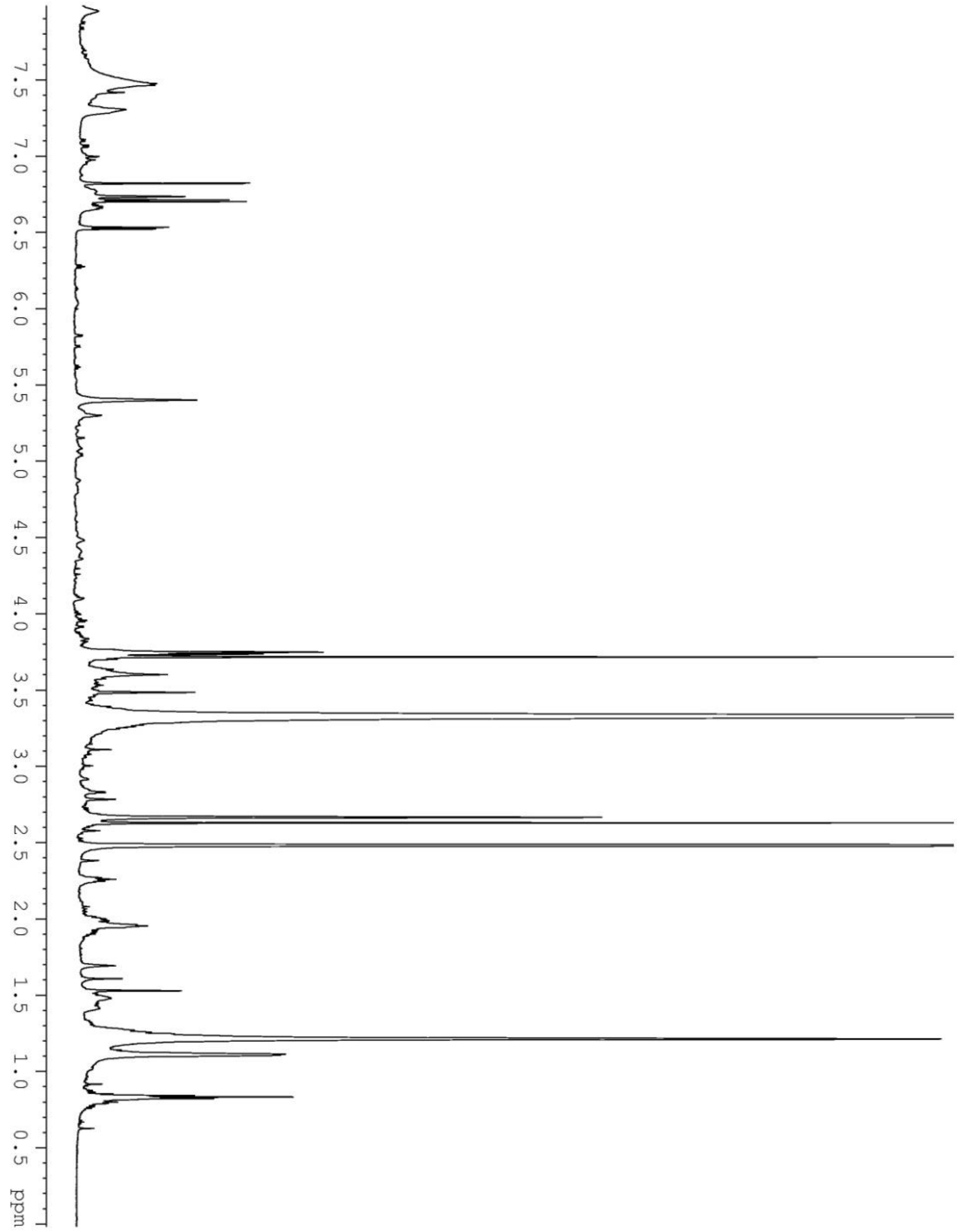

ROESY spectrum of zorrimidazolone (**2**) in DMSO

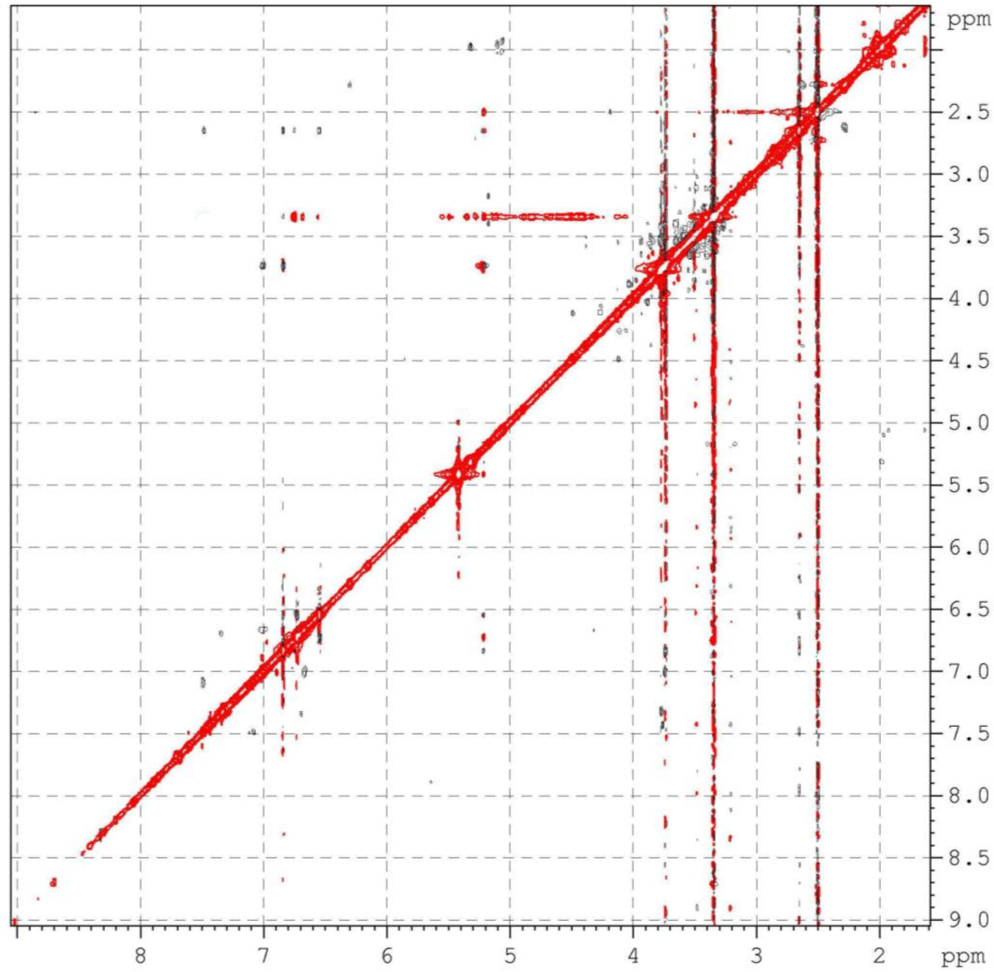

ESI mass spectrum of compound 1

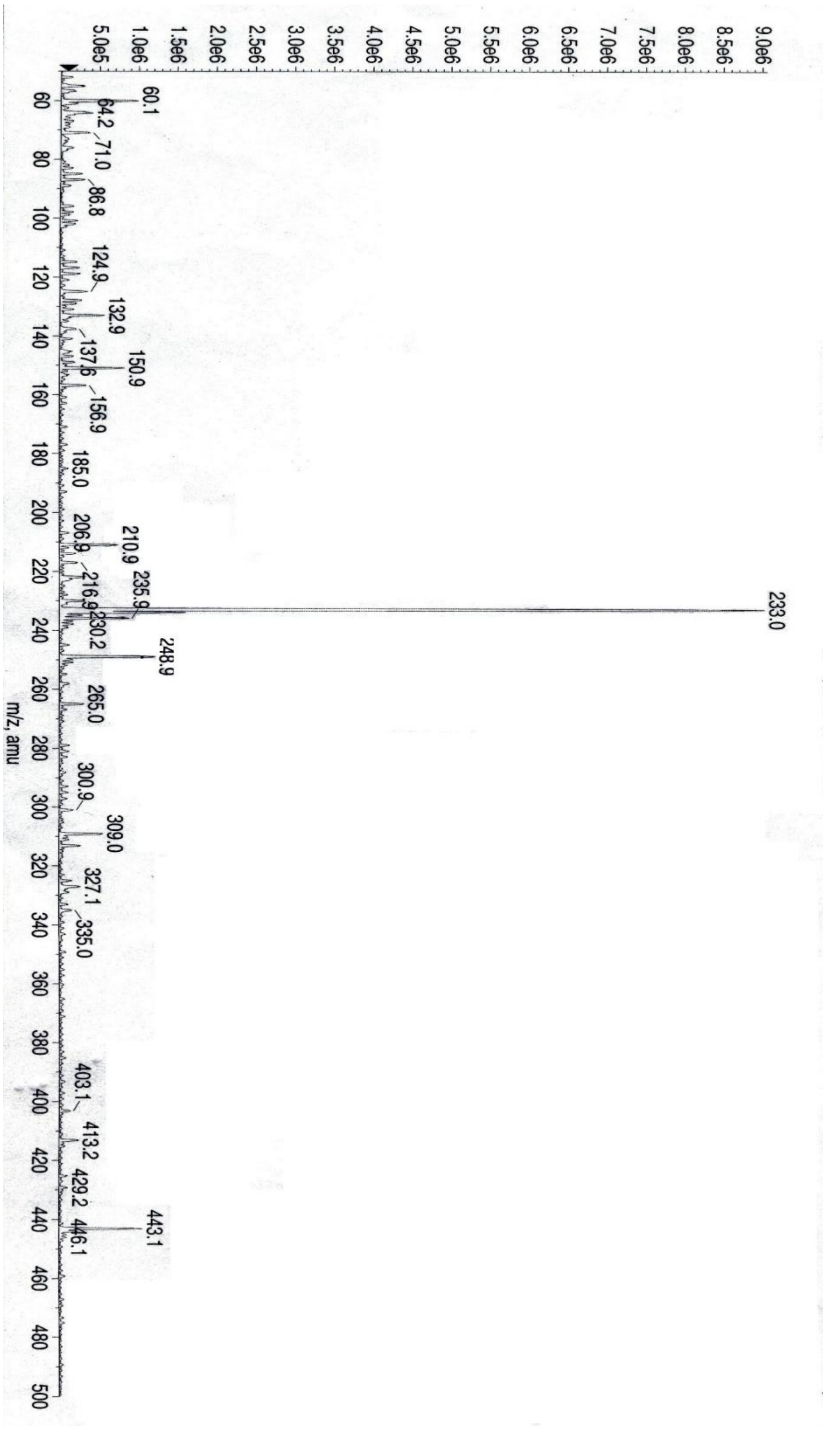

ESI mass spectrum of zorrimidazolone (**2**)

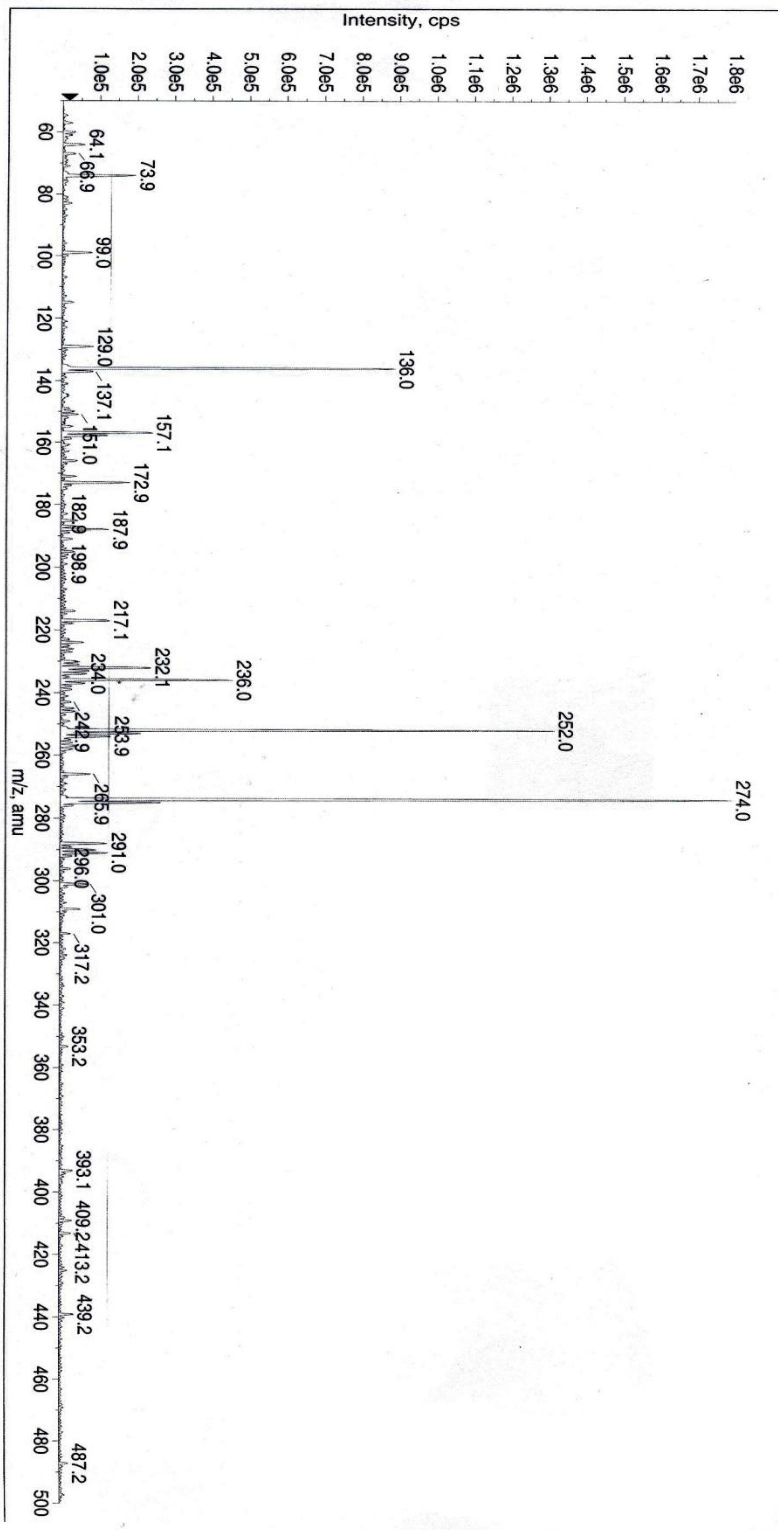

Supplement: Supplementary file 1 [file marinedrugs-09-01157-s001.pdf]
